# Survival and predictors of mortality among preterm neonates in Northern Ethiopia: A retrospective follow-up study

**DOI:** 10.3389/fped.2022.1083749

**Published:** 2023-01-13

**Authors:** Bekahegn Girma, Hailemariam Berhe, Furtuna Mekonnen, Jemberu Nigussie

**Affiliations:** ^1^ Department of Nursing, College of Medicine and Health Sciences, Dilla University, Dilla, Ethiopia; ^2^ School of Nursing, College of Health Sciences, Mekelle University, Mekelle, Ethiopia

**Keywords:** survival, predictor, mortality, preterm neonate, north, Ethiopia

## Abstract

**Background:**

In the year 2015, more than one-third of neonatal deaths caused by prematurity was recorded worldwide. Despite different kinds of efforts taken at the global and local levels to reduce neonatal mortality, it remains high with low reduction rates, especially in low- and middle-income countries like sub-Saharan Africa and South Asia. Therefore, this study aims to assess the survival status and predictors of mortality among preterm neonates.

**Methods:**

A retrospective follow-up study was conducted on randomly selected 561 preterm neonates. Data were extracted from patient records using a pretested checklist. Data entry and analysis were done using Epi-Data Version 4.4.2.1 and Stata version 14, respectively. The Cox proportional hazard regression model was fitted to identify the predictors of mortality. A hazard ratio with a 95% confidence interval (CI) was estimated and *p*-values < 0.05 were considered statistically significant.

**Result:**

The proportion of preterm neonatal deaths was 32.1% (180) with an incidence of 36.6 (95% CI: 31.6–42.4) per 1,000 person days. The mean survival time was 18.7 (95% CI: 17.7–19.9) days. Significant predictors for time to death of preterm neonates were respiratory distress syndrome [adjusted hazard ratio (AHR): 2.04; 95% CI: 1.48–2.82], perinatal asphyxia (AHR: 2.13; 95% CI: 1.32–3.47), kangaroo mother care (AHR: 0.14; 95% CI: 0.08–0.24), and gestational age (AHR: 0.85; 95% CI: 0.80–0.90).

**Conclusion:**

Preterm neonatal death is still a major public health concern. Respiratory distress syndrome, perinatal asphyxia, kangaroo mother care, and gestational age were independent significant predictors for time to death, as found in this study. Hence, priority must be given to neonates with the above illnesses and strengthen the management and care of preterm neonates.

## 1. Introduction

Neonatal mortality is a global burden that affects both developing and developed countries. Globally, approximately 2.7 million neonatal deaths were reported in the year 2015, accounting for 45·1% of under-five mortality. Prematurity was responsible for 1.05 million deaths that occurred in under-five children worldwide in 2015, of which 0.356 million deaths occurred in sub-Saharan Africa (1). In 2010, the leading cause of under-five mortality was infectious disease (64%), and prematurity-related deaths accounted for only 14% (2). Then, after 5 years, prematurity became the second leading cause of under-five mortality (3), and from 2015 until now, prematurity has been the foremost cause of under-five and neonatal mortality (1).

Despite such mortality affecting all countries of the world, there is a difference in terms of survival between developed and developing countries; neonates born in Africa have 12 times high risk of mortality compared with those born in Europe (4). Sub-Saharan Africa has a high neonatal mortality rate (27%) compared with European countries (5%) but has a low annual neonatal mortality reduction rate (2.4%) compared with the global reduction rate (3%) (5).

Preterm neonates who are born before 37 completed weeks of gestation (6) have a high risk of mortality (7, 8) and adverse health outcomes (9). Preterm birth is a major contributor to the loss of human potential (10) and to hospital admission (11). Moreover, preterm neonates who survive after the neonatal period have a high risk of neurodevelopmental and learning impairment, visual disorders, long-term cardiovascular disease, and other non-communicable diseases (12, 13). In 2010, 3.1% of disability-adjusted life years occurred because of prematurity (14).

According to the 2017 United Nations International Children's Emergency Fund (5) report, the neonatal mortality rate in Ethiopia was 27% (5). Furthermore, the 2013 UNICEF report clustered Ethiopia among the top 25 countries with high under-five mortality (3); prematurity was one of the foremost causes (15). In addition, the Ethiopian Demographic Health survey (EDHS) report indicated the trends of neonatal mortality—29% in 2016 (16) and 30% in 2019 (17). Moreover, previous studies conducted in Ethiopia showed a high neonatal mortality rate (18–21) and a low reduction rate (20).

Previous studies identified respiratory distress syndrome (RDS) (19, 22), asphyxia (18, 22, 23), sex (24–27), maternal residency (19, 28), gestational age (25, 29, 30), birth weight (18, 27, 31), neonatal sepsis (19, 23, 26), jaundice (18, 23), hyaline membrane disease (18, 23), hypothermia at admission (23), hypoglycemia (18), maternal chronic disease (19, 22), and parity (22) as a predictor of mortality for preterm neonates.

Although the above predictors have been identified, the mortality of preterm neonates continues to be high and is on an increasing trend. Furthermore, if Sustainable Development Goal 3 is to be achieved by the year 2030, conducting relevant studies on crucial topics such as neonatal mortality is essential. Such studies will also support the realization of the goal of the National Newborn and Child Survival Strategy. Therefore, this study aims to assess the survival status and predictors of mortality among preterm neonates in Northern Ethiopia.

The results of this study will help program planners, decision makers, and implementers to know the gaps in current practices of preterm neonate management, focus on the identified gaps, and take action.

## 2. Methods and materials

### 2.1. Study area and design

An institution-based retrospective follow-up study was conducted in comprehensive specialized hospitals in the Tigray region. The Tigray region is one of the nine federal administrative regions in Ethiopia. It covers an estimated area of 41,409.95 km^2^ with the capital city of Mekelle, which is approximately 781 km away from Addis Ababa, the capital city of Ethiopia. The region has an estimated total population of 5,377,144, with 2,651,167 (49.3%) males and 2,725,977 (50.7%) females. Among the total population, 159,164 are under -1-year infants. There are 2 comprehensive specialized hospitals, 15 general hospitals, 23 primary hospitals, 245 health centers, and 750 health posts in the Tigray region (32). This study was conducted in these comprehensive specialized hospitals.

The Ayder comprehensive specialized hospital (ACSH) has 45 neonatal beds in the neonatal intensive care unit (NICU) and more than 170,000 patient flows per year. The Aksum comprehensive specialized hospital provides its service to a population of over 3.6 million from the central, northwest, and western zones of the Tigray regional state. It has a total capacity of 173 beds, including 13 neonatal beds. In both hospitals, intubation, vasopressors, IV antibiotics, and gavage feeding are offered.

### 2.2. Population

In this study, the target population was all preterm neonates who were treated in the two comprehensive specialized hospitals of the Tigray region. Preterm neonates who were treated in the ACSH and Aksum comprehensive specialized hospital between February 1, 2017, and January 30, 2019, constituted the study population.

### 2.3. Eligibility criteria

Preterm neonates (born before 37 completed weeks of gestation) who were admitted to the ACSH and Aksum NICUs between February 1, 2017, and January 30, 2019, were included in this study. Preterm neonates who had incomplete medical records relating to the mainly required variables (date of birth, date of admission, outcome status, and date at which outcome was determined) were excluded.

### 2.4. Sample size determination and sampling technique

**Figure 1:**
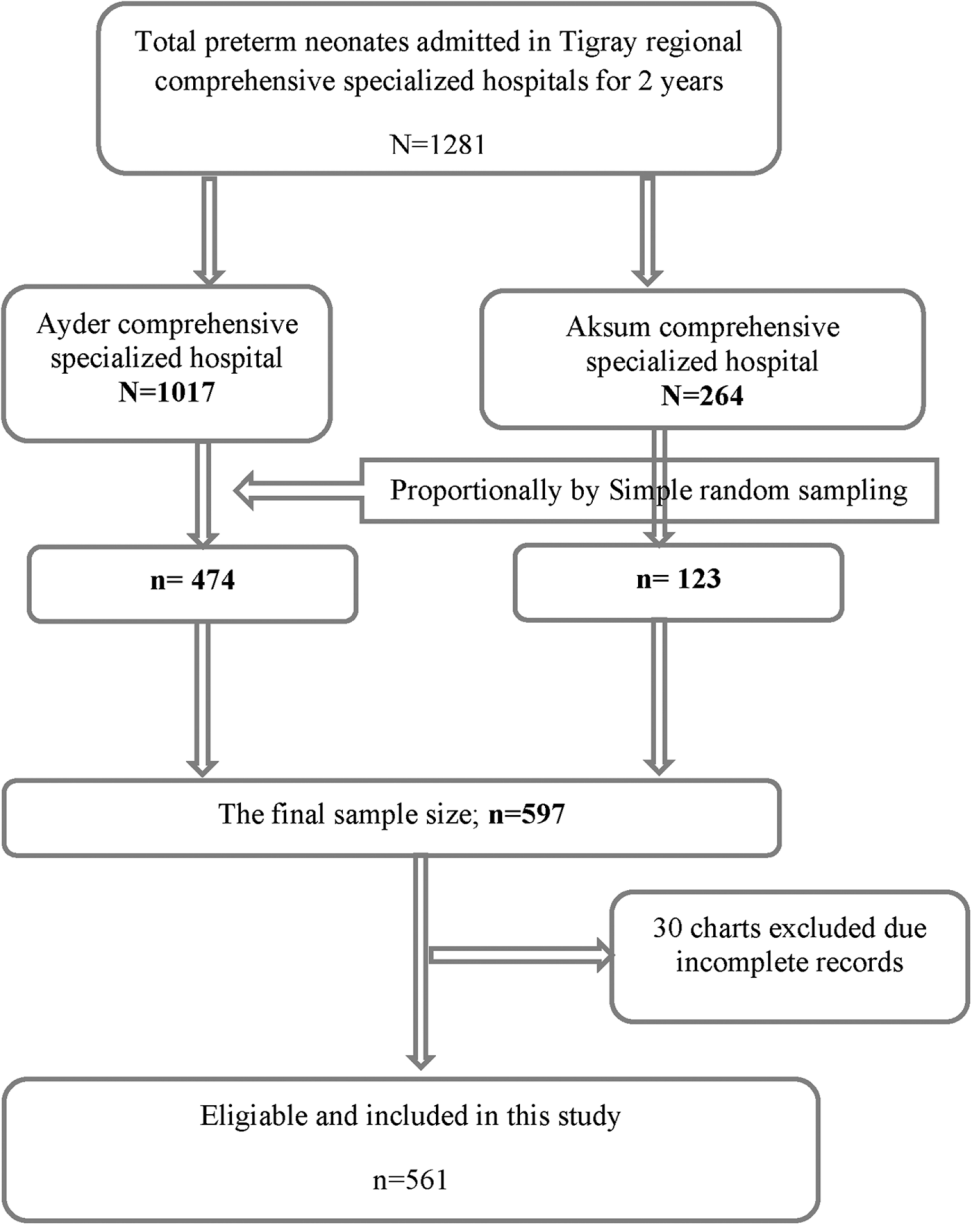
Flow diagram of study participants.

The required sample size for this study was determined by using the Stata statistical package (Cox model), version 14, and the following assumptions: a hazard ratio (HR) of 1.55 for selected covariate of interest (perinatal asphyxia, PNA) from a study done in Gondar, Ethiopia (18), a variability (SD) of 0.5, a probability of failure (death) of 0.288 (18), a margin of error of 5%, and a confidence interval (CI) of 95% to achieve 80% power. After the addition of a 5% non-response rate, the final sample size for this study was 597. A simple random sampling technique was employed. The required number of subjects were proportionally allocated for both hospitals based on their population size (Figure 1).

### 2.5. Operational definitions and measurements

The date of admission was the initial follow-up time and the follow-up was done on 28 days of life.

#### 2.5.1. Survival time

The time from admission to the event (death).

#### 2.5.2. Event

Death of preterm neonates after admission to the NICU.

#### 2.5.3. Time scale

The survival time was measured in days.

#### 2.5.4. Censored

Preterm neonates who were discharged after recovery, lost to follow-up, subjected to parental refusal to medical advice, transferred to other health institutions, and remained in the NICU after 28 days were considered censored.

#### 2.5.5. Congenital anomalies

In this study, congenital anomalies represent neural tube defects, encephalocele, anencephaly, duodenal and choanal atresia, cleft lip and palate, and gastroschisis (33).

#### 2.5.6. Respiratory distress syndrome

This condition is characterized by grunting while breathing, rapid or shallow breathing, and flaring of the nostrils (34).

#### 2.5.7. Apnea of prematurity

Respiratory pauses >20 s or pauses <20 s that are related to bradycardia (<80 beats/minute), central cyanosis, and/or oxygen saturation <85% in neonates born at <37 gestation weeks and without causal disorders that induce apnea (34).

Perinatal asphyxia: an Apgar score that remained at less than 7 (at 5 min after birth) and evidence of acute hypoxic compromise with acidemia (35).

#### 2.5.8. Neonatal jaundice

This is defined as elevated total serum bilirubin (TSB) and clinically manifests as a yellowish discoloration of the skin, sclera, and mucous membrane.

### 2.6. Data collection tool and procedures

The data were extracted from the medical records of preterm neonates who were admitted to the NICUs. A pretested checklist adapted from a study done in Addis Ababa, Ethiopia (36), was used for data collection. After permission was obtained from both hospitals, the list was prepared by investigators based on the information obtained from the NICU health management system registration book. Then, the data were scouted by some select people in this study. Finally, to fill the checklist, all obtained medical records were assessed by data collectors with supervision.

### 2.7. Data quality control

A pretest was conducted on 5% of the sample size in Mekelle general hospital. Data were collected by two trained and experienced diploma nurses and four BSc nurses, and two master's students acted as supervisors. During the data collection period, a close follow-up, monitoring, and guidance were carried out. The completeness and consistency of the data were checked daily. Before the entry of data, the checklist was patterned for tracking errors and a checklist that contained errors was excluded from the analysis.

### 2.8. Data processing, analysis, and presentation

The data were coded and entered by using Epi-Data manager version 4.4.2.1 and transferred to Stata statistical software version 14 for clearance and analysis. The Cox proportional hazard regression model was used. After Cox proportional hazard regression was done for each variable, a variable with a *p*-value <0.25 was entered for multivariate analysis. Multicollinearity was checked by using the variance inflation factor. The Cox proportional hazard regression assumption was tested by using the Schoenfeld residual test (global test). The overall goodness of model fitness was checked graphically by using the Cox Snell residual graph (Supplementary Annex S1). The overall model adequacy was checked through Harrell's C test. Lastly, after multivariate analysis, an association was expressed through the hazard ratio and statistically significant predictors were identified using 95% CI and *p*-value. The results of this study were presented by way of tables, texts, and graphs.

### 2.9. Ethics approval and consent to participate

The study protocol was evaluated and approved by the Institution of Review Board (IRB) of the College of Health Sciences, Mekelle University, and ethical clearance was obtained. A letter of cooperation was written to the chief executive managers of the ASCH and Aksum comprehensive specialized hospital. The hospital managers provided the right to access the chart of neonates. Finally, confidentiality and anonymity of the data were secured.

## 3. Result

### 3.1. Sociodemographic predictors

In this study, out of 597 preterm neonate medical charts, only 561 were complete and were included, with a 94% response rate. Out of the 561 preterm neonates, 444 (79.1%) were admitted to the ACSH, and of these, 140 (31.5%) died. Approximately 310 (55.3%) preterm neonates were males; among these, 98 (31.6%) died. Only 26 (4.6%) preterm neonates were born at home, and 12 (46.1%) of these died. A total of 406 (72.4%) moderate-to-late preterm neonates were born; among these, 95 (23.4%) died. The median gestational age at birth was 33 weeks and 6 days (IQR: 31 weeks and 3 days, 35 weeks). The median maternal age was 26 with an IQ of (22, 30) years (Table 1).

**Table 1:** Distribution of sociodemographic and obstetric-related characteristics in Northern Ethiopia, 2022 (*n* = 561).

Characteristics	Survival status		Percentage
	Death	Censored	
Place of admission			
Aksum Hospital	40	77	20.9
Ayder Hospital	140	304	79.1
Sex			
Male	98	212	55.3
Female	82	169	44.7
Place of delivery			
Home	12	14	4.6
Health center	20	46	11.8
Hospital	148	321	83.6
Gestational age in weeks			
Less than 32	85	70	27.6
Between 32 and 37	95	311	72.4
Neonatal age at admission			
Less than 24 h	170	346	92
Between 1 and 7 days	10	28	6.8
7 days and above	-	7	1.2
Residency			
Urban	96	213	55.1
Rural	84	168	44.9
Maternal age in year			
Less than 20	14	32	8.2
Between 20 and 34	148	294	78.8
35 and above	18	55	13
Parity of the mother			
Less than 2	81	200	50.1
Between 2 and 4	80	136	38.5
5 and above	19	45	11.4
Antinatal care visit			
Yes	174	372	97.3
No	6	9	2.7
Maternal corticosteroid intake			
Yes	16	65	14.4
No	164	316	85.6
Maternal hypertension			
Yes	6	13	3.4
No	174	368	96.6
Maternal sero-status			
Positive	5	13	3.2
Negative	175	368	96.8
Obstetric complications			
Yes	43	103	26
No	137	278	74
Pregnancy type			
Single	114	227	60.8
Multiple	66	154	39.2
Mode of delivery			
SVD	142	285	76.2
C/S	32	91	21.9
Instrumental	6	5	1.9
Presentation at delivery			
Cephalic	150	315	82.9
Noncephalic	30	66	17.1

SVD, spontaneous vaginal delivery; C/s, caesarian section.

### 3.2. Neonatal-related predictors

In this study, 344 (61.3%) preterm neonates had a birth weight between 1,500 and 2,500 g; 72 (20.9%) died. A total of 191 (34%) preterm neonates did not receive any feeding after delivery; 127 (66.5%) of these died. Moreover, 375 (66.8%) babies did not receive kangaroo mother care (KMC). Of these, 166 (42.3%) died. The Apgar score of 386 (68.8%) babies was known at birth. Among these, the median Apgar score at the 1st and 5th minute were 7 (IQR: 2) and 8 (IQR: 2), respectively (Table 2).

**Table 2:** Distribution of neonatal-related characteristics for preterm neonates in Northern Ethiopia, 2022 (*n* = 561).

Characteristics	Survival status		Percentage
	Death	Censored	
Birth weight in grams			
2,500 and above	5	41	87.8
Between 1,500 and 2,500	72	344	79.1
Between 1,000 and 1,500	82	153	46.4
Less than 1,000	21	23	8.7
Received kangaroo mother care			
Yes	14	186	35.6
No	166	375	64.4
Weight for gestational age			
Small	21	43	11.3
Appropriate	159	333	87.7
Large	–	5	0.89
APGAR score at birth			
Known	120	266	68.9
unknown	60	115	31.1
Having congenital anomalies			
Yes	2	14	2.8
No	178	367	97.2
Neonates with respiratory distress syndrome			
Yes	116	111	40.5
No	64	270	59.5
Neonatal jaundice			
Yes	21	44	11.6
No	159	337	88.4
Hypoglycemia diagnosed at admission			
Yes	8	50	10.3
No	172	331	89.7
Hypothermia diagnosed at admission			
Yes	78	172	44.5
No	102	209	55.5
Neonates with clinically diagnosed perinatal asphyxia			
Yes	19	12	5.5
No	161	369	94.5
Neonates with clinically diagnosed sepsis			
Yes	133	245	67.4
No	47	136	32.6
Newborns with anemia			
Yes	5	14	3.4
No	175	367	96.6
Newborns diagnosed with thrombocytopenia			
Yes	8	5	2.3
No	172	376	97.7
Newborns with apnea of prematurity			
Yes	16	8	4.3
No	164	373	95.7
Neonates with necrotizing enterocolitis			
Yes	9	3	2.1
No	171	378	97.9

### 3.3. Preterm birth–related predictors

Only 41 (7%) preterm neonates did not have medical and surgical complications. Among those who had such complications, 227 (40.5%), 378 (67.4%), 13 (2.3%), and 12 (2.1%) of them had RDS, sepsis, thrombocytopenia, and necrotizing enterocolitis (NEC), respectively (Table 3).

**Table 3:** Bivariable and multivariate Cox proportional hazard regression output for predictors to the time to death of preterm neonates in Northern Ethiopia, between February 1, 2017, and January 30, 2022 (*n* = 561).

Variables	Categories	Survival status		CHR (95% CI)	AHR (95% CI)
		Death	Censored		
Kangaroo mother care	Yes	14	172	0.12 (0.07–0.21)	0.14 (0.08–0.24)**
	No	166	209	1	
Gestational age	–			0.79 (0.74–0.84)	0.85 (0.80–0.90)**
Maternal corticosteroid exposure	Yes	16	65	1	
	No	164	316	1.76 (1.05–2.93)	1.18 (0.70–2.00)
Respiratory distress syndrome	Yes	116	111	2.65 (1.96–3.60)	2.04 (1.48–2.82)**
	No	64	270	1	
Perinatal asphyxia	Yes	19	12	2.46 (1.52–3.96)	2.13 (1.32–3.47)*
	No	161	369	1	
Anemia	Yes	3	14	0.42 (0.13–1.31)	0.38 (0.12–1.21)
	No	177	367	1	
Apnea of prematurity	Yes	16	8	2.14 (1.28–3.58)	1.45 (0.85–2.47)
	No	164	373	1	
Hypoglycemia	Yes	78	172	0.49 (0.24–0.99)	0.94 (0.45–1.95)
	No	102	209	1	
Congenital anomalies	Yes	2	14	0.36 (0.09–1.47)	1.76 (0.43–7.18)
	No	178	367	1	

CHR, crude hazard ratio; AHR, adjusted hazard ratio.

* Significant (*p*-value **< **0.01).

** Significant (*p* < 0.05).

### 3.4. Maternal- and obstetric-related predictors

Out of 561 preterm neonates, 50% were born to mothers who had a parity of less than 2, and 546 (97.3%) were born to mothers who had antenatal care (ANC) follow-up. A total of 460 (86.2%) babies were born to mothers who had three and more ANC visits. A majority (85.6%) of preterm neonates were born to mothers who had not taken corticosteroids before delivery; of these, 164 (34.2%) died. A total of 146babies (26%) were born to mothers who had obstetric complications; among these, 40 (27.4%) were born to those who suffered antepartum hemorrhage (Table 1).

### 3.5. Overall survival function

**Figure 2:**
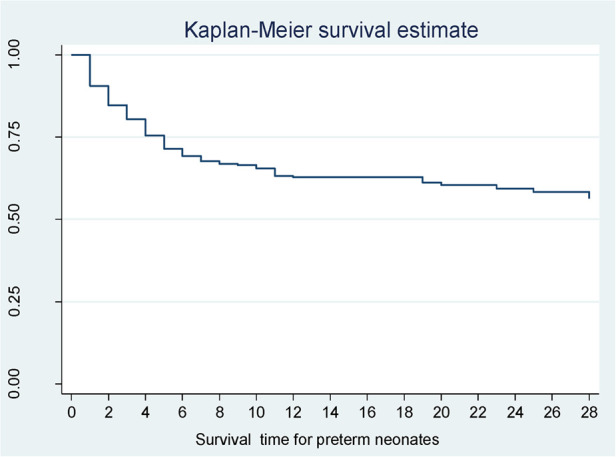
Overall survival function for preterm neonates in Northern Ethiopia, between February 1, 2017, and January 30, 2022 (*n* = 561).

**Figure 3:**
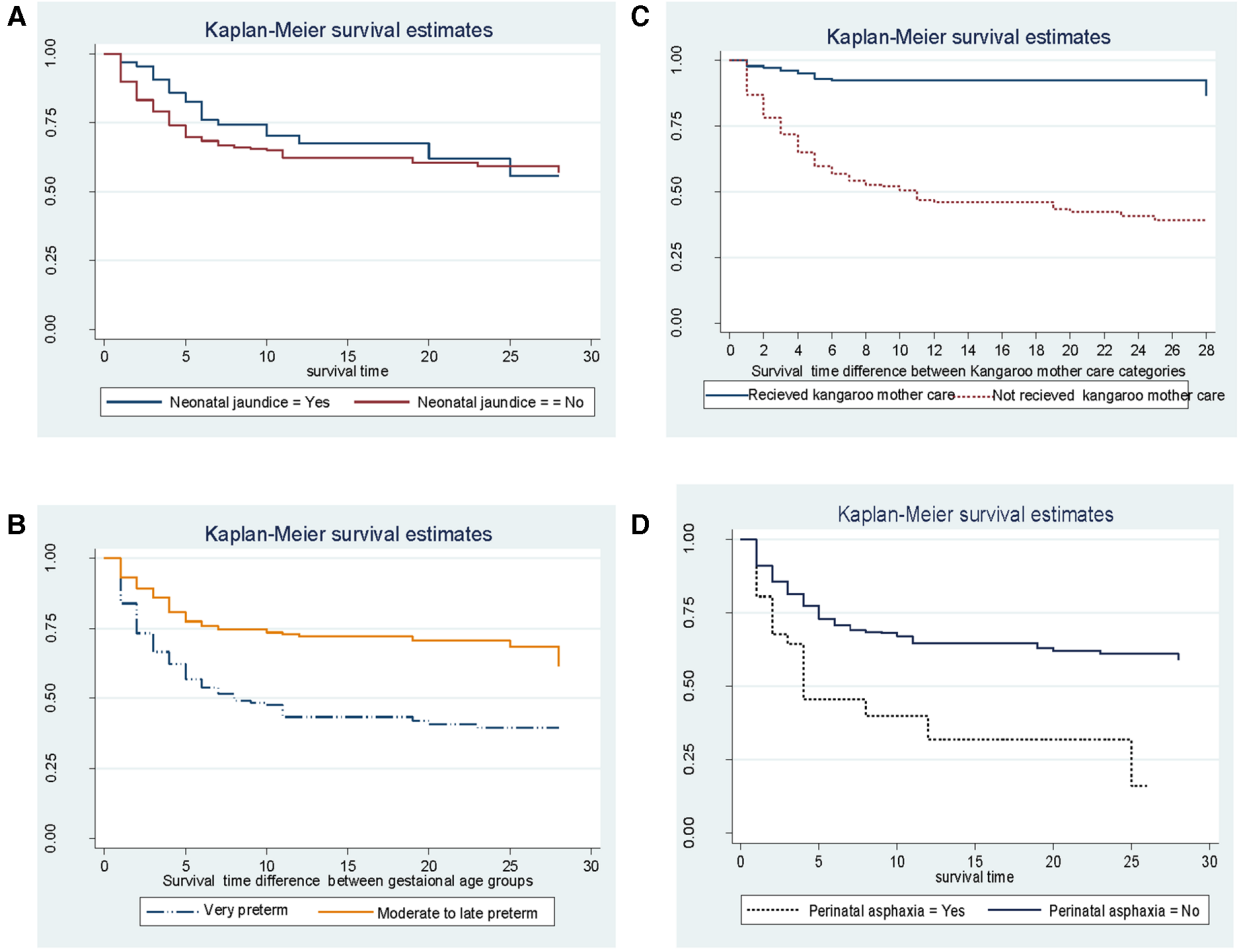
Kaplan–Meier estimated survival difference for selected covariates (**A–D**) of preterm neonates in Northern Ethiopia, between February 1, 2017, and January 30, 2019, and 2022 (*n* = 561).

According to Kaplan–Meier survival estimates, 160 (88.9%) deaths occurred in the first week of admission, of which 53 (33.1%) died within the first 24 h of admission. In this study, the survival function vs. survival time was a decreasing step function (Figure 2). The survival probabilities for preterm neonates at the end of the first day, 7th day of admission, 14th day of admission, 21 days, and at the end of 28 days of admission were 90.5%, 67.7%, 62.8%, 60.4%, and 56.4%, respectively. Kaplan–Meier survival function estimation graphs were prepared for categorical covariates to observe survival differences (Figure 3).

### 3.6. Survival status of preterm neonates

**Figure 4:**
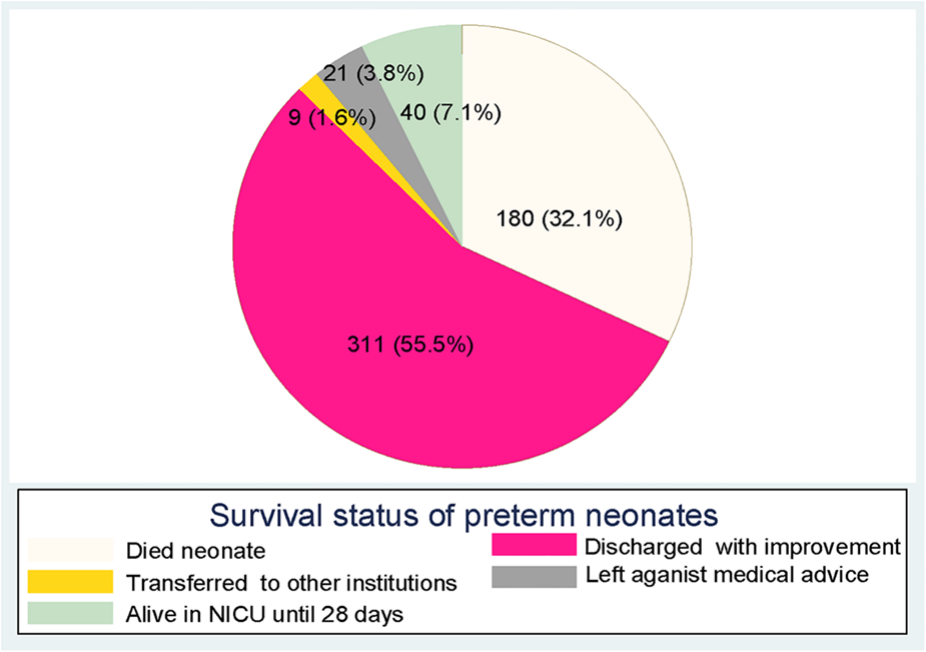
Survival status of preterm neonates at the end of follow-up in Northern Ethiopia, between February 1, 2017, and January 30, 2019, and 2022 (*n* = 561).

In this study, 180 (32.1%) preterm neonates died during the follow-up period (Figure 4). The median length of follow-up was 6 days. The total person-day-observations were 4,917 days. The overall incidence of mortality for preterm neonates was 36.6 (95% CI: 31.6, 42.4) per 1,000 person days. Furthermore, the overall mean survival time of preterm neonates was 18.8 (95% CI: 17.7–19.9) days.

### 3.7. Predictors for time to death of preterm neonates

Bivariable and multivariate Cox proportional hazard regression were fitted to identify predictors for the time to death of preterm neonates. Variables that had a *p*-value <0.25 in the bivariable analysis were entered into the multivariate analysis. Findings from the bivariable analysis showed that respiratory distress syndrome, birth weight, apnea of prematurity, anemia, perinatal asphyxia, kangaroo mother care, congenital anomalies, gestational age, maternal corticosteroid intake, and hypoglycemia at admission were significantly associated with the time to death of preterm neonates. Before multivariate analysis, the global test was checked and it did not violate the assumption; the overall global test = 0.53. The model adequacy test (Harrell's C test) was 0.8 (80%). Multicollinearity was checked by VIF and it was 1.1. In multivariate analysis, four variables, respiratory distress syndrome, perinatal asphyxia, gestational age, and kangaroo mother care, were identified as independent predictors for the time to death of preterm neonates.

Preterm neonates having respiratory distress syndrome were twice [adjusted hazard ratio (AHR): 2.04; 95% CI: 1.48–2.82] more likely to die compared with their counterparts. Neonates having perinatal asphyxia were twice (AHR: 2.13; 95% CI: 1.32–3.47) more likely to die compared with their counterparts in the comparison group. As the gestational age increased in 1 week, the death rate decreased by 15% (AHR = 0.85; 95% CI: 0.80–0.90). Preterm neonates who received kangaroo mother care were 86% (AHR: 0.14; 95% CI: 0.08–0.24) less likely to die compared with their counterparts (Table 3).

## 4. Discussion

This study aimed to assess the survival status and predictors of mortality among preterm neonates admitted in the comprehensive specialized hospitals located in Northern Ethiopia, Tigray region. The incidence of mortality and the mean survival time for preterm neonates were 36.6 (95% CI: 31.6–42.4) per 1,000 person-day-observations and 18.8 days, respectively. RDS, PNA, KMC, and gestational age were identified as independent significant predictors for the time to death of preterm neonates.

The mean survival time of preterm neonates was lower than that in a study done in the University of Gondar (UOG) comprehensive specialized hospital, Ethiopia, which was 20.4 (22). This difference might be due to a high mortality rate in this study. Furthermore, the mean survival time of preterm neonates in this study was also lower than that in a study conducted in Iran, which was 43.0 (29). This variation might be due to differences in the length of follow-up. In a study conducted in Iran, preterm neonates were followed up until discharge with a maximum hospital stay of 105 days. However, in this study, neonates were followed up until 28 days, which made the survival time lower than that in the above study finding.

Preterm neonates who had RDS during hospital stay had increased hazards or risks of death compared with their counterparts. This finding was in line with a study conducted at Jimma University specialized hospital in Ethiopia (23). Another study that was conducted in Ethiopia also supported this finding (22). These study agreements might be attributed to the fact that the main cause of respiratory distress syndrome in preterm neonates is hyaline membrane deficiency, and preterm neonates who have RDS mostly develop acute complications such as pulmonary hemorrhage, apnea of prematurity, and intraventricular hemorrhage (IVH), which increase the risk of mortality (37).

Neonates who had perinatal asphyxia were more likely to die compared with their counterparts. This finding was consistent with that of studies done at the UOG comprehensive specialized hospital (18, 22) and Jimma University specialized hospital (23). This similarity might be attributed to the same levels of quality care provision for asphyxiated preterm neonates because all hospitals were comprehensive specialized hospitals. Also, perinatal asphyxia is one of the leading causes of neonatal mortality in Ethiopia (16), and perinatal asphyxia has the potential to cause damage to all organs of the body, including the brain and kidneys, and to impair gas exchange, in addition to causing organ immaturity in preterm neonates that facilitates neonatal mortality. Moreover, this similarity might be attributed to perinatal asphyxia causing severe complications such as hypoxic ischemic encephalopathy (HIE) and IVH, which have a high level of incidence in preterm neonates (15).

Preterm neonates who received KMC had less risks of mortality, and this result was similar to that of the study conducted in the UOG comprehensive specialized hospital (18). This similarity might be due to the majority of preterm neonates not receiving kangaroo mother care, as found in both studies, and KMC also protects neonates from infection, effectively treat hypothermia, improve gastrointestinal function and cardiorespiratory stability, and initiate/encourage breastfeeding. One systematic review and meta-analysis also supported this finding (38). In view of the important benefits of KMC, the World Health Organization (WHO) has strongly recommended its use as a package to treat preterm neonates (39).

In this study, as noted previously, it was found that when the gestational age increased by 1 week, the risk of mortality decreased by 15%. This finding was in line with that of a study conducted at the University of Gondar, Ethiopia (18). Also, this finding was supported by studies conducted in Ethiopia (23), Iran (29), and East Africa (30). Clinical evidence also supports this finding. The link between gestational age and mortality could be traced to the fact that organ immaturity leads to higher mortality; the more preterm, the more organ immaturity.

Because of the retrospective nature of the study design, an incomplete recording of data was a major limitation of this study.

## 5. Conclusion

The incidence of preterm neonatal mortality was high in this study. The most critical period for preterm neonatal mortality was the first week of admission, especially the first 24 h. The mean survival time of preterm neonates in this study was found to be lower than that in previous studies. Respiratory distress syndrome, perinatal asphyxia, gestational age, and kangaroo mother care were identified as predictors for the time to death of preterm neonates. To decrease the risk of mortality, health professionals should strengthen their follow-up regimens for very preterm neonates and neonates who have PNA and RDS. Moreover, health professionals should make the implementation of kangaroo mother care more stringent, since our study was retrospective study institutional related factors were not assessed. Therefore, we recommend prospective studies.

## Data Availability

The original contributions presented in the study are included in the article/Supplementary Material, further inquiries can be directed to the corresponding author.
