# Supplementary material for: Survival and predictors of mortality among preterm neonates in Northern Ethiopia: A retrospective follow-up study
Source: Front Pediatr. 2023 Jan 13;10:1083749. doi: 10.3389/fped.2022.1083749 (PMC9880159; doi:10.3389/fped.2022.1083749)
Supplement: Supplementary file 1 [file Datasheet1.docx]

|   **Annexe *1*:** Cox Snell residual graph to test the goodness of the Cox model fitness |
| --- |
